# Supplementary material for: Runway: A novel, forward-screen method of analyzing catwalk gait analysis data
Source: PLoS One. 2025 Sep 24;20(9):e0325831. doi: 10.1371/journal.pone.0325831 (PMC12459821; doi:10.1371/journal.pone.0325831)
Supplement: Attachment — Submitted filename: Response to Reviewers 050225.docx [file pone.0325831.s003.docx]

Please find attached the revised version of our manuscript entitled “**Runway: A Novel, Forward-Screen Method of Analyzing Catwalk Gait Analysis Data".** We greatly appreciate the feedback offered on our previous submission and have taken steps to address any critiques or concerns raised.

In summary, we are greatly appreciative of the feedback supplied by both reviewers, and have incorporated their critiques into this revised manuscript. Please let us know if there remain any errors or unaddressed issues in this revised copy, and we will make the changes as soon as possible.

Below is a summary of the review feedback provided, annotated with our response.

***The authors should support their statements with references in the Introduction much more often (e.g.: line 37, 44, 64). To find the place of this method among the existing ones the authors should cite more than 16 papers.***

We agree that more citations are necessary in our instruction, and have added additional ones to further support our statements.

***Regeneration of the motor system takes long time in most of the experiments resulting in the growth and gaining weight of the animals (in rats it is significant). How does the package handle this phenomenon? If it is not able to do so yet, how will this correlation introduced?***

We agree that this is a significant factor in animal recovery post injury, and have added additional language starting at line 296 to address how our method can incorporate these variables.

***Both step length and step duration vary in healthy animals within wide ranges affecting the “style” of the gait. How does the dataset manage to handle these parameters?***

We agree that these factors are critical components, and have addressed them along with further discussing the importance of considering baseline performance with edits starting at line 166

***Video-based semi-automatic gait analysing systems (e.g. DeepLabCut ) should be mentioned in the Introduction (with references) and they should be compared to the present method in the Discussion.***

We strongly agree, and have included multiple reference to DeepLabCut and similar methods throughout the introduction and discussion sections.

***Is there any possibility in the future to adopt this method to other systems different from CatWalk?***

Yes, this is highly possible, and we have added edits starting at lines 73 and 296 to address this.
